# HIV-1 infection of microglial cells in a reconstituted humanized mouse model and identification of compounds that selectively reverse HIV latency

**DOI:** 10.1007/s13365-017-0604-2

**Published:** 2017-12-18

**Authors:** George N. Llewellyn, David Alvarez-Carbonell, Morgan Chateau, Jonathan Karn, Paula M. Cannon

**Affiliations:** 1 Department of Molecular Microbiology and Immunology, Keck School of Medicine, University of Southern California, Los Angeles, CA USA; 2 Department of Molecular Biology and Microbiology, School of Medicine, Case Western Reserve University, Cleveland, OH USA

**Keywords:** HIV latency, Epigenetic silencing, Humanized mice, Microglial cell

## Abstract

Most studies of HIV latency focus on the peripheral population of resting memory T cells, but the brain also contains a distinct reservoir of HIV-infected cells in microglia, perivascular macrophages, and astrocytes. Studying HIV in the brain has been challenging, since live cells are difficult to recover from autopsy samples and primate models of SIV infection utilize viruses that are more myeloid-tropic than HIV due to the expression of Vpx. Development of a realistic small animal model would greatly advance studies of this important reservoir and permit definitive studies of HIV latency. When radiation or busulfan-conditioned, immune-deficient NSG mice are transplanted with human hematopoietic stem cells, human cells from the bone marrow enter the brain and differentiate to express microglia-specific markers. After infection with replication competent HIV, virus was detected in these bone marrow-derived human microglia. Studies of HIV latency in this model would be greatly enhanced by the development of compounds that can selectively reverse HIV latency in microglial cells. Our studies have identified members of the CoREST repression complex as key regulators of HIV latency in microglia in both rat and human microglial cell lines. The monoamine oxidase (MAO) and potential CoREST inhibitor, phenelzine, which is brain penetrant, was able to stimulate HIV production in human microglial cell lines and human glial cells recovered from the brains of HIV-infected humanized mice. The humanized mice we have developed therefore show great promise as a model system for the development of strategies aimed at defining and reducing the CNS reservoir.

## Introduction

Reservoirs of HIV persist in the body despite antiretroviral therapy (ART) (Barton et al. 2016; Boritz et al. 2016; Brew et al. 2015; Katlama et al. 2013). The reservoir includes populations of latently infected cells that can be reactivated and restart an infection when ART is interrupted. Most studies of HIV latency have focused on the central memory T cells found in the peripheral circulation (Brew et al. 2015; Siliciano and Siliciano 2013; Spivak and Planelles 2016); however, the CNS contains a significant reservoir that is distinct from the T cell compartment that has often been neglected (reviewed in (Fois and Brew 2015; Hellmuth et al. 2015)).

Direct evidence for HIV infection of the brain has come from autopsy samples (Desplats et al. 2013; Thompson et al. 2011), with HIV DNA detected in astrocytes, perivascular macrophages, and microglia (Churchill et al. 2006; Thompson et al. 2011). Although astrocytes show infection rates up to 19% (Churchill et al. 2009), they produce little to no infectious virus (Gorry et al. 2003; Messam and Major 2000), suggesting that the source of persisting HIV in the CNS is instead the microglia or perivascular macrophages. Primary human microglia can be readily infected by HIV ex vivo (Garcia-Mesa et al. 2017; Jordan et al. 1991), and recent studies have shown that they are much more permissive to infection than macrophages from other tissues (Cenker et al. 2017).

Persistence of HIV in the CNS may be facilitated by sub-optimal drug penetration across the blood-brain barrier, although there is little evidence for a high level of ongoing active replication in the CNS in well-suppressed patients or the emergence of drug-resistant strains (Bednar et al. 2015; Dahl et al. 2014; Gianella et al. 2016; Schnell et al. 2011). Virus also rebounds in the CNS after ART interruption, analogously to lymphoid tissues (Gianella et al. 2016). We therefore favor the hypothesis that HIV persists in the CNS due primarily to the establishment of latent HIV infections, especially in the microglial cell population, which is highly permissive for HIV infection (Cenker et al. 2017). Curing HIV will therefore require strategies that remove, disable, or suppress all latent HIV reservoirs, including those specific to the CNS (Brew et al. 2015; Fois and Brew 2015; Garrido and Margolis 2015).

Studies of HIV infection in the CNS are challenging, and have relied historically on post-mortem samples or on cell line models that may not fully recapitulate in vivo systems. Although primate SIV infection models have provided important insights about CNS infection, (Zink et al. 1999), the presence of Vpx in SIV, which counteracts the myeloid restriction factor SamHD1 and leads to enhanced infection of perivascular macrophages and microglial cells, means that some findings from the SIV models may not be translatable to HIV. There is therefore a pressing need to develop better animal models of HIV infection in the CNS that not only support infection of relevant human cell types, such as human perivascular macrophages and microglia, but also recapitulate features of the HIV life cycle, including the development of latency during ART.

Humanized mice are being explored as a readily accessible model for HIV CNS infection (Honeycutt et al. 2015). Early approaches included the direct injection of HIV-infected monocyte-derived macrophages into the brains of immunodeficient mice (Persidsky et al. 1996; Poluektova et al. 2002, 2004; Potula et al. 2005; Tyor et al. 1993). Although this can result in high levels of engraftment around the site of injection, the mice only survived up to 4 weeks post-injection, and the trauma caused by the injection itself induced potentially confounding levels of inflammation (Honeycutt et al. 2015). In contrast, systemic injection of human HSC into immune-deficient strains, such as NSG mice, creates humanized mice that support long-term engraftment of human cells in the blood and lymphoid tissues. Unfortunately, only low numbers of human cells have been observed in the brains of these mice by histology and qPCR (Honeycutt et al. 2016; Dash et al. 2011). When humanized mice are infected with HIV, p24+ cells can be detected in the brain (Gorantla et al. 2010; Honeycutt et al. 2016), and neuronal damage, inflammation, and increased mouse microglia proliferation have been reported (Dash et al. 2011; Boska et al. 2014; Gorantla et al. 2010), mimicking aspects of AIDS-related dementia seen in untreated patients. Although some of the human cells in humanized mouse brains have been reported to express the microglia marker Iba1 (Asheuer et al. 2004), none of the published studies have specifically demonstrated HIV-infected human microglia.

In the present study, we characterized the human cells present in the brains of NSG mice transplanted with human HSC and identified human cells expressing markers of microglia. Using specific conditioning regimens, up to 10% of the microglia in the brains of these animals were of human origin and could be readily infected by HIV in vivo*.* This is a critical first step to investigate whether latency can develop in the microglial cell population in vivo. Our previous studies of immortalized human microglial cells have shown that latency can readily develop in microglial cells due to the imposition of epigenetic restrictions (Alvarez-Carbonell et al. 2017; Garcia-Mesa et al. 2017). In order to develop tools to study latency in the humanized mouse model, we used these cell models to identify compounds that can potently and selectively reverse latency in microglial cells. Intriguingly, after isolation of the human microglial cells from the mice, viral reactivation was achieved using the monoamine oxidase (MAO) inhibitor phenelzine, suggesting that a subset of these cells may harbor latent proviruses.

## Results

### Strategy for developing a humanized mouse model to study HIV latency

Our strategy to repopulate the brains of immune-deficient NSG mice with human microglial cells was based on prior studies showing that depletion of CNS myeloid cells occurs following treatment with radiation (Eglitis and Mezey 1997), or by exposure of CD11b-HSVTK transgenic mice to intracerebroventricular ganciclovir (GCV) (Varvel et al. 2012), allows repopulation of such microglia-depleted brains by mouse peripheral monocytes. In the studies of Varvel et al. (2012), GCV depletion allowed the brains to become repopulated with bone marrow-derived monocytes that expressed high levels of CD45 and CCR2 and, upon entry into the brain, expressed the sentinel microglial marker Iba1. Although the infiltrating monocytes were two times more numerous and morphologically distinct from resident microglia, they became uniformly distributed throughout the brain, and had an overall distribution and behavior that was remarkably similar to that of microglia. In addition, work by Asheuer et al. (2004) demonstrated that the repopulating cells could also be derived from transplanted human bone marrow cells. Adapting and simplifying this method for use with HIV, we reasoned that NSG mice reconstituted with human hematopoietic stem cells would also contain cells that could differentiate into a microglial phenotype in the brain and subsequently support infection by HIV.

### Identification and quantification of human microglia in humanized NSG mice

**Fig. 1:**
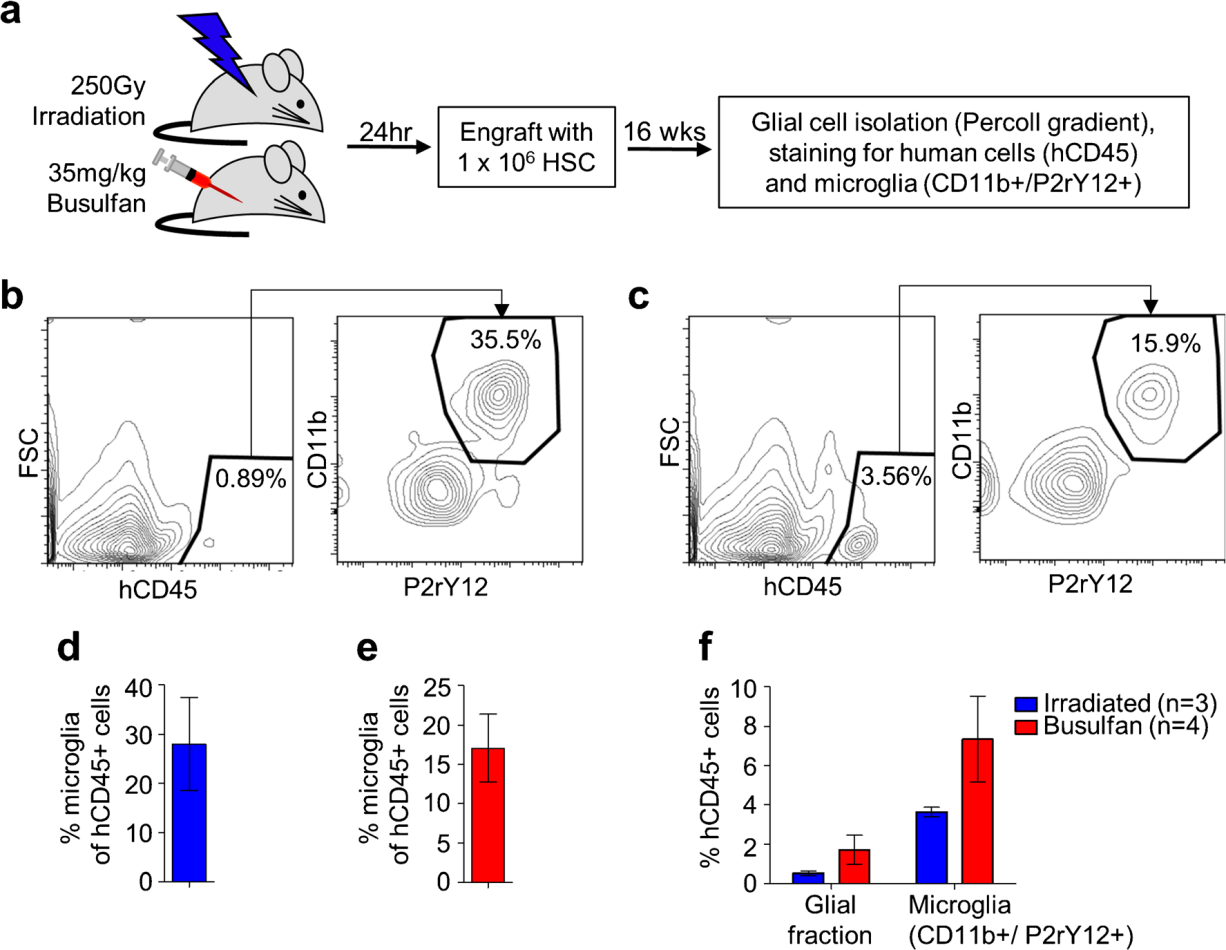
Human microglia in the brains of humanized mice. **a** Experimental scheme to create humanized mice using either irradiation or busulfan conditioning. At necropsy, the total glial fraction was isolated using a Percoll gradient, and the human cells and microglia in that fraction identified by flow cytometry using indicated markers. **b** Representative flow cytometry analysis of human microglia (hCD45+/CD11b+/P2rY12+) in an irradiated mouse. **c** Representative flow cytometry plot analysis of human microglia in a mouse conditioned with busulfan. **d** Quantification of human microglia in *n* = 3 irradiated mice. **e** Quantification of human microglia in *n* = 4 busulfan-conditioned mice. **f** Comparison of levels of human cell (hCD45+) contribution in the glial fraction, and the total microglial population, in irradiated versus busulfan-conditioned mice

Humanized NSG mice were created by standard procedures using total body irradiation to condition adult mice, followed by transplantation with up to 10^6^ human CD34+ HSC (Holt et al. 2010; Wang et al. 2015) (Fig. 1 a). At the same time, we also evaluated an alternate conditioning regimen based on the chemotherapeutic agent, busulfan, since this has been reported to increase the frequency of donor HSC-derived microglia present in the brains of mice undergoing transplantation with mouse HSC (Wilkinson et al. 2013). The CD34+ cells used to generate these mice were isolated from a single source to eliminate human donor cell variation.

At 16-week post-transplantation, mice were euthanized, perfused with PBS to deplete contaminating blood cells from the brain, and the glial fraction was then isolated from brain tissue using a Percoll gradient. This fraction was immunostained for a human-specific marker (hCD45), as well as the microglial markers CD11b and P2RY12 (Fig. 1b–e) (Bennett et al. 2016; Butovsky et al. 2014). P2RY12 is a more specific marker for microglia than CD11b, which is also expressed on macrophages, but we observed that almost all the cells expressing either marker were both P2RY12^+^ and CD11b^+^. We found both an increase in the overall frequency of human cells in the brains of busulfan-conditioned mice compared to the irradiated mice, and a corresponding increase in the frequency of human cells in the total CD11b^+^ P2RY12^+^ fraction, which includes both mouse and human microglia (Fig. 1e).

### HIV infection of microglia in the brain of humanized mice

**Fig. 2:**
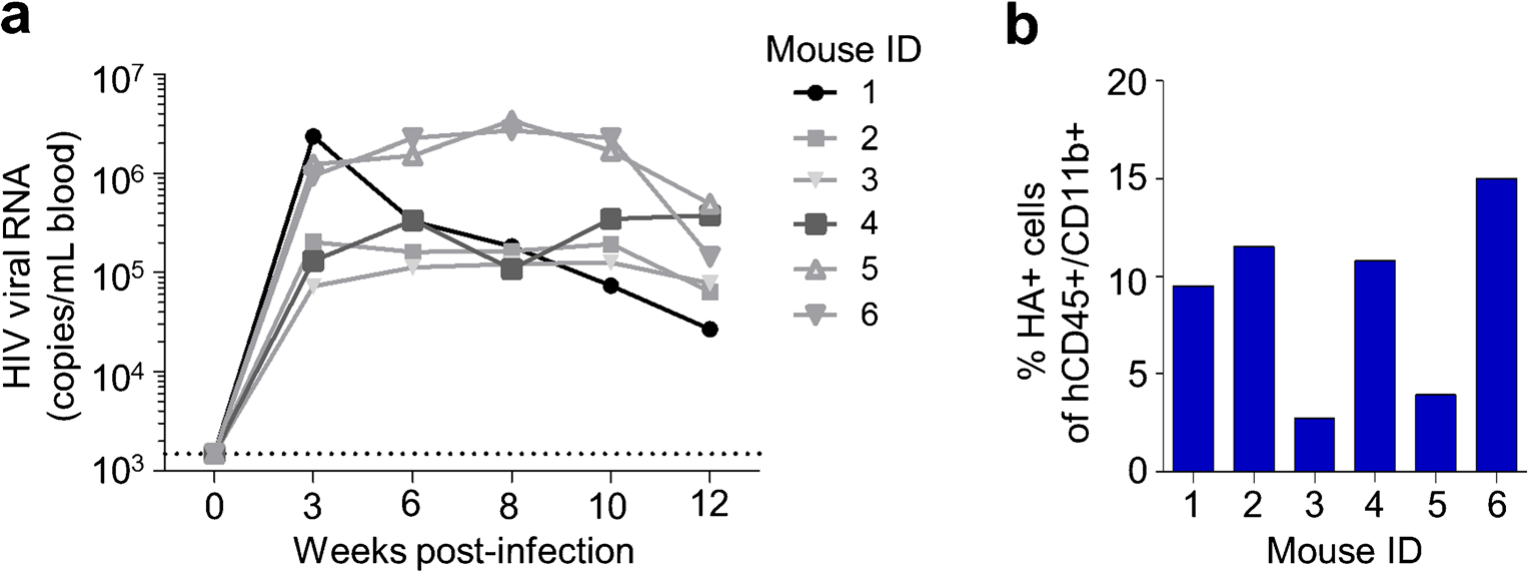
HIV infection of microglia. **a** Neonate-engrafted humanized mice were infected with HIV strain JRCSF-HA (*n* = 6). Virus levels in the blood were measured over time. Dotted line represents limit of detection of assay = 1.5 × 10^3^. **b** After 12 weeks post-infection, glial cells from individual mice were analyzed by flow cytometry to quantify the frequency of HIV infection (HA+) in cells expressing markers of human microglia (hCD45+ CD11b+)

To determine whether the human microglia in the brains of humanized mice could be infected by HIV in vivo, we challenged five humanized mice with an R5-tropic strain of HIV, JRCSF-HA. This virus contains a surface expressed hemagglutinin (HA) epitope-tagged protein that can be used to identify productively infected cells by flow cytometry. Viremia was monitored in the blood of all animals over 12 weeks (Fig. 2a), at which point the mice were necropsied and the glial cells isolated and stained for hCD45, CD11b, and HA. We observed that 2–15% of the hCD45+ CD11b+ cells were also positive for HA, indicating the presence of productively HIV-infected microglia (Fig. 2b).

### Identification of selective inducers of HIV proviral latency

Although significant progress has been made in our understanding of the mechanisms promoting HIV latency in T cells, the mechanisms that could contribute to latency in microglia are less well understood (Alvarez-Carbonell et al. 2017). To identify genes involved in maintaining HIV latency in microglia, we performed a genome-wide shRNA library screen using CHME-5/HIV cells. CHME-5/HIV cells are a rat microglial cell line that contains a single integrated copy of a defective reporter HIV genome, in which Nef has been replaced by a short-lived GFP reporter (d2EGFP). The HIV genome is latent, but can be reactivated to express GFP by agents such as TNF-α or trichostatin A (Wires et al. 2012).

**Fig. 3:**
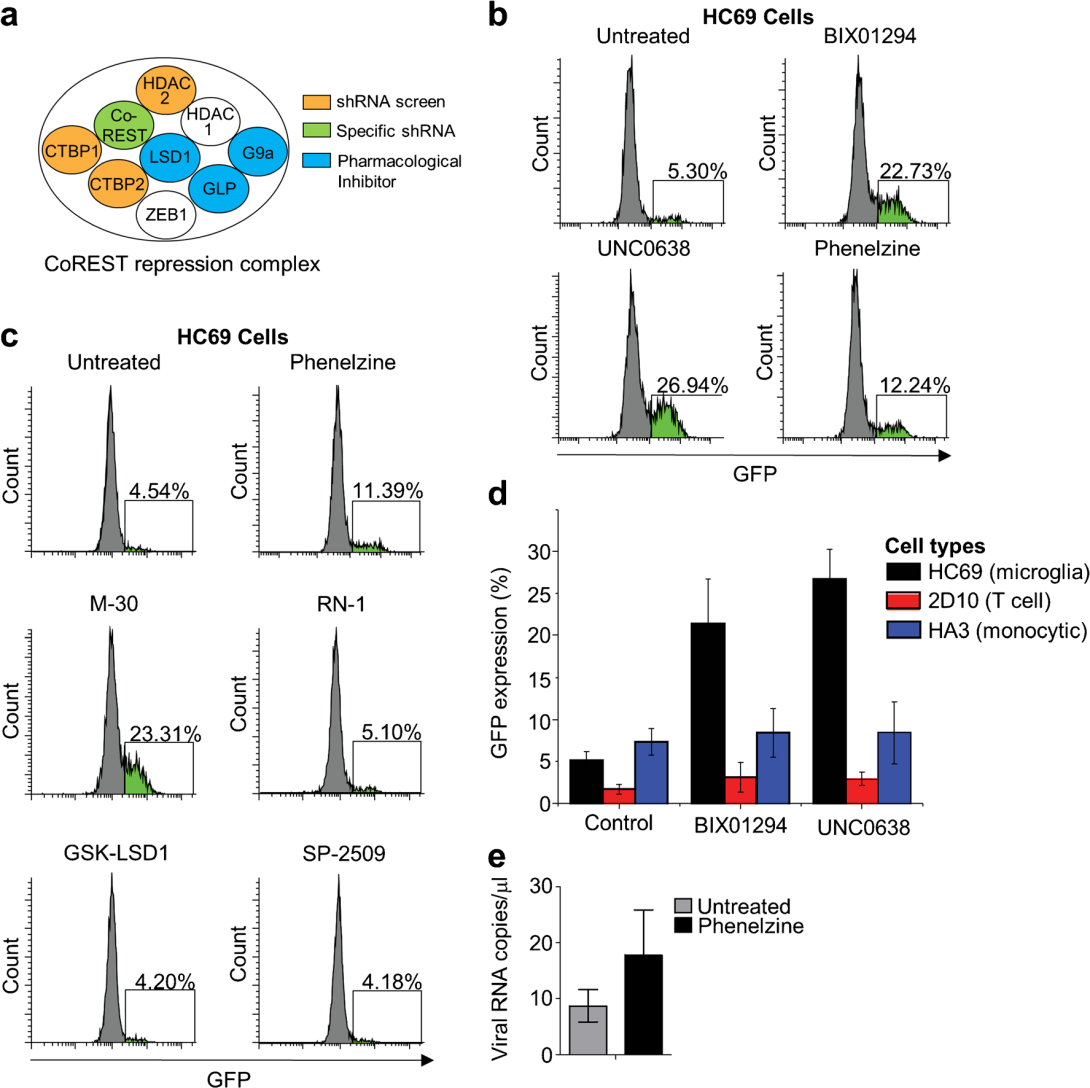
Inhibition of G9a/EHMT2, GLP/EHMT1, or MAO promotes HIV emergence from latency in microglia. **a** CHME-5/HIV cells were subjected to genome-wide shRNA screening to identify shRNAs that increased HIV (GFP) expression. Members of the CoREST repressor complex are shown, with the three shRNA target genes with high level hits indicated (orange). The role of CoREST in latency maintenance was also confirmed by specific shRNA knockdown (green). Other members of the complex were analyzed in HC69 cells (see below) using pharmacological inhibitors (blue). **b** HIV emergence from latency in human microglial HC69 cells, detected by GFP^+^ expression at 16 h post-treatment using inhibitors BIX01294 (G9a inhibitor), UNC0638 (GLP/G9a inhibitor), and phenelzine (LSD1 and MAO inhibitor). In the flow cytometry plots, GFP^+^ cell populations are colored green, and the percentage of GFP-expressing cells is indicated. **c** To determine whether phenelzine activation of HIV latency is due to LSD1 or MAO, HC69 cells were treated with LSD1 inhibitors (phenelzine, RN-1, GSK-LSD1, or SP-2509) or MAO inhibitors (phenelzine or M-30) and analyzed for GFP expression. **d** Activation of HIV from HC69 cells (microglia latency—black bars), 2D10 cells (T cell latency—red bars), or HA3 cells (monocytic latency—blue bars), treated with the indicated drugs. Error bars represent the standard deviation of three or more experiments. **e** Glial cells from five individual JRCSF-HA-infected mice were cultured with or without phenelzine for 2 days, and HIV copies in the culture supernatants measured by qRT-PCR. Error bars represent standard error of the mean

The shRNA screen used a genome-wide lentiviral library from Cellecta, combined with systems biology classifications of the hits, as previously described (Li et al. 2016; Nguyen et al. 2017). Briefly, the CHME-5/HIV cells were transduced with the shRNA library vectors, and GFP^+^ cells were selected by cell sorting. The target genes of the shRNAs leading to reactivation determined by sequencing (Li et al. 2016; Nguyen et al. 2017). As summarized in Fig. 3a, members of the CoREST repression complex, including HDAC2, CTBP1, and CTBP2, were highly ranked “hits” in the screen. These candidates were confirmed by specific shRNA-mediated knockdown of the CoREST protein and its associated co-factors in CHME-5/HIV cells.

To confirm the role of the CoREST repression complex in maintaining HIV latency in microglial cells, we used our recently developed model of HIV latency in human microglia cells (Hμglia/HIV), clone HC69 (Alvarez-Carbonell et al. 2017; Garcia-Mesa et al. 2017). These cells were derived from immortalized primary human microglia and contain a latent reporter HIV construct that expresses GFP after proviral reactivation. HC69 cells were treated with pharmacological inhibitors that target G9a and GLP, two essential members of the CoREST complex. As shown in Fig. 3b, there was latency-reversing activity by BIX01294 (22.73% GFP^+^), which targets G9a/EHMT2 (Shi et al. 2003) and UNC0638 (26.94% GFP^+^), which targets the GLPL/G9a (EHMT1/EHMT2) heterodimer (Vedadi et al. 2011). Both compounds by themselves were able to increase GFP expression significantly above basal levels (5.3% GFP^+^).

To determine whether the epigenetic latency-reversing factors that we identified were specific for microglia, we tested BIX01294 and UNC0638 on 2D10 cells (Pearson et al. 2008), a latently infected Jurkat T cell line carrying a similar GFP reporter construct. Neither compound reactivated GFP in these cells (Fig. 3d), however, both compounds are weak latency-reversing agents in primary T cells and patient cells (Nguyen et al. 2017).

We also tested the monoamine oxidase (MAO) inhibitor phenelzine. Phenelzine was drawn to our attention because, in addition to it MAO inhibition activity, it has been reported to inhibit another member of the CoREST complex, LSD1 (KDM1) (Culhane et al. 2010; Sakane et al. 2011). We found that phenelzine was a moderate latency-reversing agent in HC69 cells (12.24% GFP^+^) (Fig. 3b).

To evaluate whether the effect of phenelzine on HIV reactivation was due to its anti-LSD1 (KDM1) activity or its anti-MAO activity, we tested a series of highly specific MAO inhibitors or LSD-1 (KDM1) inhibitors. M-30, a specific inhibitor of MAO, reactivated GFP in HC69 cells (23.31% GFP^+^) while in contrast, the specific LSD1 (KDM1) inhibitors RN-1, GSK-LSD1, and SP-2509 had no effect (Fig. 3c). This indicates that the phenelzine-mediated reactivation of latent HIV is due primarily to its anti-MAO activity, possibly due to the increased NF-κB phosphorylation stimulation by phenelzine (Chung et al. 2012). Thus, MAO inhibition clearly provides a potent and selective means to reactivate HIV in microglial cells.

### Human microglia isolated from HIV-infected humanized mice can be used to evaluate latency reversal drugs

The latency-reversing agents that are active in the microglia cell line models of HIV latency can potentially be used as tools to stimulate HIV production from human microglia infected in vivo in humanized mice. For an initial study, we used phenelzine. Although this compound is not as potent as some of the other MAO inhibitors that we have identified, it is nonetheless highly selective for microglial cells and is an FDA-approved antidepressant with proven blood-brain barrier penetration.

We isolated the total glial cell population from 5 JRCSF-infected humanized mice and cultured them ex vivo, with or without phenelzine, for 2 days. HIV released into the supernatant was measured by qRT-PCR of viral RNA. As shown in Fig. 3e, there was an approximately twofold increase in the amount of HIV released from phenelzine-treated cells compared to untreated cells.

An unusual feature of the reversal of HIV latency is that the induction of Tat leads to an “all or nothing” induction of transcription. When latency is reversed, intermediate levels of HIV expression in those cells are never seen. Therefore, increases in HIV expression invariably correlate with an increase in the number of cells producing HIV (Jadlowsky et al. 2014; Mbonye and Karn 2017; Nguyen et al. 2017). Thus, the enhanced viral production seen after treatment of the glial cell population with phenelzine is consistent with reversal of latency in a subset of the infected microglial cell population (Fig. 3e).

## Discussion

### HSC-reconstituted mice harbor human microglia in their brains

A major cell type in the CNS that supports productive infection by HIV is the microglia, a macrophage-like cell that functions as the primary immune cell of the brain. To facilitate studies of both productive and latent infections of microglia that could contribute to the persistence of HIV in the CNS during ART, we have begun to characterize the human cells present in the brains of HSC-transplanted, HIV-infected humanized mice. We readily detected human cells with markers of microglia in these humanized mice, based on co-expression of CD11b and the more specific microglial marker, P2RY12 (Butovsky et al. 2014). Furthermore, in HIV-infected animals, we detected HIV-infected cells in this fraction (mean 9% of human microglia).

Despite the macrophage-like properties of microglia, fate-tracking studies have shown that they develop from an embryonic lineage, yolk sac erythro-myeloid progenitors, that are distinct from HSC, which derive from the aorta-gonad-mesonephros region (Medvinsky and Dzierzak 1996). Accordingly, it has been debated whether or not HSC can give rise post-natally to microglia (Ginhoux et al. 2013). However, mouse-to-mouse transplant experiments have shown that donor HSC produce cells that are phenotypically indistinguishable from microglia in mice whose resident microglia have been depleted by radiation, chemotherapy, or through microglia-specific expression of a suicide gene (Capotondo et al. 2012; Derecki et al. 2012; Sergijenko et al. 2013; Varvel et al. 2012). Furthermore, injection of human HSC into radiation-treated immune-deficient mice populates the brain with human cells expressing microglial markers (Asheuer et al. 2004). These observations also fit with findings from clinical trials using genetically modified autologous HSC to treat neurological diseases such as ALD, where conditioning allows microglia derived from the engineered HSC to provide enzymatic trans-complementation and clinical benefit (Cartier et al. 2009).

Our characterization of humanized mice also supports the idea that transient depletion of endogenous mouse microglia allows repopulation by human HSC-derived cells. Conditioning using busulfan, which depletes endogenous microglia more than irradiation, nearly doubles microglial cell engraftment in the brains of the mice. This also provides a simple technical advance that makes the humanized mouse model a more effective and practical approach to studies of HIV infection in the brain.

### MAO inhibitors specifically reactivate latent HIV in microglia

We are currently using humanized mice to study whether latently infected microglia can persist in mice treated with ART, and to evaluate whether any such reservoir could be perturbed by compounds that specifically reactivate latent HIV in microglia. We have previously reported that distinct pathways are involved in the activation of latent HIV in microglia, including the TLR3 pathway (Alvarez-Carbonell et al. 2017). We used a shRNA screen in a cell line model of HIV latency to identify microglia-specific pathways based on the CoREST repressor complex. Pharmacological inhibition of key epigenetics-modifying enzymes in this complex (EHMT1 and EHMT2) led to the specific reactivation of latent HIV in microglial cells, but not in cell line models of latent HIV in T cells or monocytes. These results are consistent with previous reports that EHMT2 is an important regulator of HIV latency in microglial cell lines (Le Douce et al. 2012; Marban et al. 2007). While phenelzine also acts as a weak inhibitor of the CoREST complex protein LSD-1 (KDM1), we found that it is its ability to inhibit MAO that reversed latency in microglial cells. We then studied whether other MAO inhibitors, such as M-30, could also reactivate HIV and found that they are indeed potent and selective reactivating agents in microglial cells.

The reactivation of latent HIV by specific drugs is being considered as an adjunct to ART that could reduce the latent reservoir through a “shock and kill” approach and thereby allow an HIV cure. Although the major focus in such studies has been on drugs that can activate latent T cells, latent reservoirs in the CNS are likely to become a major barrier to curing HIV (Brew et al. 2015; Darcis et al. 2017; Katlama et al. 2013; Siliciano and Siliciano 2013). Phenelzine is FDA-approved as an antidepressant and has proven blood-brain barrier penetration. Therefore, phenelzine, along with other MAO inhibitors, should become useful tools to reverse HIV latency in the humanized mouse model.

### Modeling HIV latency in microglial cells using HSC-reconstituted humanized mice

We also evaluated the ability of phenelzine to stimulate HIV production from microglia harvested from HIV-infected humanized mice and found that it was able to increase HIV production in these cells approximately twofold. In this experiment, the difference in levels of virus released from unstimulated and phenelzine-stimulated cells was likely minimized because the cells used for this experiment were from non-ART-treated mice, which increases the background of HIV production in the untreated cell control. Although we have not yet formally ruled out that this effect of phenelzine is due instead to enhanced production of HIV from productively infected cells, work in cell models has shown that induced cells produce HIV Tat and therefore have a uniformly maximal induction of HIV transcription. Thus, increases in HIV production after latency reversal invariably reflect increases in the number of productive cells (Jadlowsky et al. 2014; Kim et al. 2011; Mbonye et al. 2013; Nguyen et al. 2017). The phenelzine data therefore indicates that a subset of microglia recovered from the reconstituted mice are latently infected and can be reactivated by this drug.

Going forward, we plan to study the effect of various latency reactivators, including pro-inflammatory molecules and inhibitors of repressor complexes, on HIV emergence from latency in microglia harvested from ART-treated animals. We are also expanding the ex vivo studies using sensitive intracellular RNA detection methods to more rigorously demonstrate latency in this system.

### Modeling HIV-associated neurocognitive disorders

HIV-1 replication in the CNS is probably initiated from invading monocytes and then spreads to microglial cells and astrocytes within the brain parenchyma (Churchill et al. 2009; Cosenza et al. 2002; Fischer-Smith et al. 2004; Liu et al. 2004; Takahashi et al. 1996; Wiley 2003; Wiley et al. 1986). Definitive evidence that HIV replicates in macrophages within the CNS comes from the observation that HIV-associated dementia (HAD) patients harbor macrophage-tropic HIV-1 variants that grow selectively in the CNS (Gorry et al. 2002; Peters et al. 2004; Rossi et al. 2008; Schnell et al. 2011). A consequence of HIV-1 replication in longer-lived cell types in the brain, such as microglial cells, is that virus in the cerebrospinal fluid (CSF) decays more slowly than virus found in the peripheral circulation after the initiation of therapy (Schnell et al. 2009).

An important contribution to HIV-1 neuropathology is the combined neurotoxic effects of viral proteins and exaggerated inflammatory responses. In vitro studies have demonstrated the toxic effects of the viral proteins gp120 (Kaul and Lipton 2006) and Tat (El-Hage et al. 2011; Li et al. 2009) on neurons. Both in vitro and in vivo studies have shown that immune-activated, HIV-infected, brain-infiltrating macrophages, and resident microglia, also release high levels of neurotoxic cytokines such as TNF-α and IL-1β (Kaul and Lipton 2006). Cytokine release is also a response to HIV proteins, since exposure of macrophages to intact HIV-1 virions or gp120 induced IL-1β release independently of productive infection (Cheung et al. 2008; Herbein et al. 1994; Merrill et al. 1989).

Importantly, neuronal dysfunction does not correlate with the number of HIV-infected cells or viral antigens in CNS (Glass et al. 1995; Masliah et al. 1997), but rather with elevated inflammatory cytokine levels. Elevated TNF-α mRNA levels in microglia and astrocytes (Glass et al. 1995; Wesselingh et al. 1997) and high levels of IL-1β and TNF-α are seen in the CNS of patients with HAD (Brabers and Nottet 2006; Epstein and Gendelman 1993). Similarly, increased IL-6 and IL-8 levels have also been reported in the brains of HIV-1 infected patients (Breen et al. 1990), and several studies show that gp120 also induces IL-6 expression in mixed cultures of human primary brain cells (Yeung et al. 1995) and murine primary mixed glial cell cultures (Kong et al. 1996). A central role for IL-6 in gp120-induced neuroinflammation has been demonstrated using a rat model (Schoeniger-Skinner et al. 2007), where intrathecal administration of gp120 induced the expression of IL-6, TNF-α, and IL-1β.

It is notable that the inflammatory signals correlated with HAND in well-suppressed patients are the same signals that induce latent HIV in microglial cell models (Alvarez-Carbonell et al. 2017). The development of the humanized mouse models described here offers an exciting opportunity to investigate the inter-relationship between HIV latency and chronic inflammatory on the development of neuropathology in vivo.

## Methods

### Cell lines

Cell lines CHME-5/HIV (microglia), HC69 (microglia), 2D10 (T cell), and HA3 (monocytic) are models of HIV latency, containing a GFP reporter in place of *gag* in an HIV proviral clone, and expressing GFP only when stimulated (Alvarez-Carbonell et al. 2017; Garcia-Mesa et al. 2017; Pearson et al. 2008; Wires et al. 2012). CHME-5/HIV cells were cultured in DMEM plus 5% FBS (ThermoFisher Scientific, Carlsbad, CA), HC69 cells in DMEM plus 1% FBS, 2D10, and HA3 cells in RPMI plus 10% FBS (ThermoFisher Scientific).

### Creation of humanized mice

NOD Cg-*Prkcd* ^*scid*^ *Il2rgtm1* ^*Wjl*^ */*SzJ (NSG) were conditioned using sub-lethal radiation doses of 150 Gy (neonates) or 250 Gy (adults), or treated with 35 mg/kg busulfan (adults) (Alpha Aesar, Haverhill, MA). After 4 h (irradiation) or 24 h (busulfan), the mice were injected with 1 × 10^6^ CD34+ cells, isolated from human fetal liver, as previously described (Holt et al. 2010; Wang et al. 2015). Human cell engraftment levels in the blood were measured in animals from 8 weeks of age using an antibody mix containing human-specific antibodies: anti-hCD3-PE (UCHT1) anti-hCD4-FITC (RPA-T4) and anti-hCD45-PerCP (2D1) (BD Biosciences, San Jose, CA).

### HIV-1 virus production and infection of humanized mice

The HIV-1 proviral clone, JRCSF-HA, was generated by inserting an HA epitope-tagged HSA protein into the *vpr* open reading frame of JRCSF (Koyanagi et al. 1987) and is similar to the NL4-3-HSA-HA clone previously published (Ali and Yang 2006). Virus stocks of JRCSF and JRCSF-HA were generated by transient transfection of HEK 293T cells (ATCC, Manassas, VA), using 18 μg of plasmid in a 10-cm plate, essentially as described (Cannon et al. 1994). Virus titer (infectious units/mL) was determined by infection of Ghost(3)X4/R5 cells, obtained through the NIH AIDS Reagent Program, Division of AIDS, NIAID, NIH, from Drs. Vineet N. Kewalramani and Dan R. Littman (Morner et al. 1999), as previously described (Cecilia et al. 1998). Humanized mice between ages 12 to 20 weeks, engrafted with at least 30% human CD45+ cells in the blood, of which at least 10% were CD3+/CD4+, were infected with JRCSF or JRCSF-HA, using 500 μL intraperitoneal injections containing 5 to 10 × 10^5^ infectious units.

### HIV-1 qRT-PCR

HIV-1 RNA was extracted from either 50 μL of mouse blood, or 100 μL cell culture supernatants, using a Qiagen Viral RNA Isolation Kit according to the manufacturer’s instructions (Qiagen, Hilden, Germany). qRT PCR was performed using the Taqman RNA-to-CT One Step Kit, according to the manufacturer’s instructions (Applied Biosystems, Foster City, CA). Primers used were LTR-F: 5′-GCCTCAATAAAGCTTGCCTTGAG-3′ and LTR-R: 5′-GGCGCCACTGCTAGAGATTTTC-3′, with a FAM-TAM probe sequence: 5′-AAGTAGTGTGTGCCCGTCTGTTRTKTGACT-3′ (Applied Biosystems). Cycling conditions used were 1 cycle of 45 °C for 35 min, then 43 cycles of 95 and 68 °C for 1 min each. Standards were 10-fold dilutions of an in-house titered NL4-3 virus stock. Limit of detection (LOD) of the assay is 10 copies, which corresponds to 1500 copies/mL mouse blood or 500 copies/mL cell culture supernatant.

### Glial cell isolation and flow cytometry

Mice were anesthetized with avertin and cardiac perfused with 30–50 mL PBS to remove lymphocytes and macrophages from the brain. Glial cells were isolated essentially as described (Moussaud and Draheim 2010). Briefly, brains were minced and collagenase/dispase (Roche, Basil, Switzerland) treated for 30 min at 37 °C and then DNAse treated (Roche). Cells were washed with PBS and resuspended in 20% isotonic Percoll (in Hank’s balanced saline solution (HBSS) (ThermoFisher Scientific), with HBSS layered on top, and centrifuged for 20 min at 200 g. The interphase containing myelinated cells was discarded and the pelleted fraction (glial cells) washed with HBSS and immunostained with anti-human CD45-PerCP (2D1) (BD Biosciences), anti-CD11b-PE (M1/70) (Biolegend, San Diego, CA) and anti-P2RY12 (Abcam, Cambridge, United Kingdom). For P2RY12 (unconjugated), donkey anti-rabbit Alexa Fluor 488 (ThermoFisher Scientific) was used as a secondary antibody. Flow cytometry analyses were performed using a FACSCanto II (BD Biosciences), with compensation samples created using BD CompBeads (BD Biosciences). Data were analyzed using FlowJo software V7.6.5 (Treestar, Ashland, OR).

### Unbiased human shRNA screening

CHME-5/HIV cells were subjected to genome-wide shRNA screening using a human shRNA library packaged in VSV-G pseudotyped lentiviral vectors (Cellecta Inc., Mountain View, CA). The lentiviral library comprises 82,500 shRNAs targeting 15,439 mRNA sequences and carries a puromycin-resistance cassette. Transduced cells were selected in puromycin (2 μg/mL), and GFP-positive cells, which indicate HIV reactivation, selected by FACS. Puromycin-resistant/GFP^+^ cells were expanded to 1 × 10^7^ cells and subjected to an additional two rounds of GFP^+^ sorting. After the third round of purification/expansion, when nearly 50% of the cells were constitutively GFP^+^, genomic DNA was isolated from 4 × 10^6^ cells and used as a template for nested PCR using primers that recognize sequences flanking the unique bar-code sequences in the lentiviral vectors, and which are unique for each shRNA. High throughput deep sequencing was performed on the nested PCR-amplified product and the sequenced bar-codes de-convoluted to their shRNA sequences using the Decipher Deconvolution software (Cellecta Inc.). The shRNAs were then ranked based on their abundance, and classified by Ingenuity Pathway Analysis™.

### Transduction of CHME-5/HIV cells with specific shRNA vectors

10^5^ CHME-5/HIV cells were transduced with VSV-G-pseudotyped lentiviral vectors expressing scrambled or CoREST specific shRNAs. The shRNAs were inserted in the pLKO.1 backbone and comprised the scrambled non-silencing GIPZ lentiviral shRNAmir control and CoREST shRNA RHS4533-NM_015156, obtained from Thermo Scientific Open Biosystems. Three days after transduction, drug-resistant cells were selected in medium containing puromycin (2 μg/mL) for at least 7 days. Cell viability and GFP expression were assessed by FACS and fluorescence microscopy, as described below.

### Analysis of HIV reactivation in latently infected cell lines

Quantification of GFP expression was performed by flow cytometry analysis using a LSR Fortessa instrument, the FACSDiva software (BD, NJ) for data collection, and the WinList 3D software for data analysis. Prior to analysis, CHME-5/HIV and HC69 cells (adherent) were trypsinized, collected, and resuspended in 300 μL of cold PBS, while the suspension cells 2D10 and HA3 were centrifuged, and pellets resuspended in 300 μL of PBS. Drug treatments of HIV-latently infected CHME-5/HIV, HC69, HA3, and 2D10 cells were typical for 16 h, with the following concentrations: 4 μM BIX01294 (Sigma-Aldrich B9311), 3 μM UNC0638 (Sigma-Aldrich U4885), 500 μM phenelzine (Sigma-Aldrich P6777), 100 μM M-30 (Sigma-Aldrich SML0128), 100 μM RN-1 (Tocris 4977), 100 μM GSK-LSD1 (Sigma-Aldrich SML1072), and 2 μM SP-2509 (Cayman Chemical 15487).

### Ex vivo activation of HIV in glial cell fraction

Glial cells (5 × 10^5^) isolated from HIV-infected humanized mice were cultured in DMEM-F/12 (ThermoFisher Scientific), 10% FBS and penicillin/streptomycin, with or without 5 μM phenelzine (Sigma). After 2 days, HIV was quantified from the supernatants via RNA extraction and qRT-PCR, as described above.
